# Manual Therapy Reduces Pain Behavior and Oxidative Stress in a Murine Model of Complex Regional Pain Syndrome Type I

**DOI:** 10.3390/brainsci9080197

**Published:** 2019-08-10

**Authors:** Afonso S. I. Salgado, Juliana Stramosk, Daniela D. Ludtke, Ana C. C. Kuci, Daiana C. Salm, Lisandro A. Ceci, Fabricia Petronilho, Drielly Florentino, Lucineia G. Danielski, Aline Gassenferth, Luana R. Souza, Gislaine T. Rezin, Adair R. S. Santos, Leidiane Mazzardo-Martins, William R. Reed, Daniel F. Martins

**Affiliations:** 1 Coordinator of Integrative Physical Therapy Residency–Philadelphia University Center, Londrina 86020-000, Paraná, Brazil; 2 Experimental Neuroscience Laboratory (LaNEx), University of Southern Santa Catarina, Palhoça 88137-270, Santa Catarina, Brazil; 3 Postgraduate Program in Health Sciences, University of Southern Santa Catarina, Palhoça 88137-270, Santa Catarina, Brazil; 4 Laboratory of Neurobiology of Inflammatory and Metabolic Processes, Graduate Program in Health Sciences, Health Sciences Unit, University of South Santa Catarina, Tubarão 88704-900, Santa Catarina, Brazil; 5 Laboratory of Neurobiology of Pain and Inflammation, Department of Physiological Sciences, Centre of Biological Sciences, University Federal of Santa Catarina, Florianópolis 88040-900, Santa Catarina, Brazil; 6 Postgraduate Program in Neuroscience, Center of Biological Sciences, Federal University of Santa Catarina, Florianópolis 88040-900, Santa Catarina, Brazil; 7 Department of Physical Therapy, School of Health Professions, University of Alabama at Birmingham, Birmingham, AL 35294-1212, USA

**Keywords:** chronic pain, Complex Regional Pain Syndrome, manual therapy, osteopathy, oxidative stress

## Abstract

Complex regional pain syndrome type I (CRPS-I) is a chronic painful condition. We investigated whether manual therapy (MT), in a chronic post-ischemia pain (CPIP) model, is capable of reducing pain behavior and oxidative stress. Male Swiss mice were subjected to ischemia-reperfusion (IR) to mimic CRPS-I. Animals received ankle joint mobilization 48h after the IR procedure, and response to mechanical stimuli was evaluated. For biochemical analyses, mitochondrial function as well as oxidative stress thiobarbituric acid reactive substances (TBARS), protein carbonyls, antioxidant enzymes superoxide dismutase (SOD) and catalase (CAT) levels were determined. IR induced mechanical hyperalgesia which was subsequently reduced by acute MT treatment. The concentrations of oxidative stress parameters were increased following IR with MT treatment preventing these increases in malondialdehyde (MDA) and carbonyls protein. IR diminished the levels of SOD and CAT activity and MT treatment prevented this decrease in CAT but not in SOD activity. IR also diminished mitochondrial complex activity, and MT treatment was ineffective in preventing this decrease. In conclusion, repeated sessions of MT resulted in antihyperalgesic effects mediated, at least partially, through the prevention of an increase of MDA and protein carbonyls levels and an improvement in the antioxidant defense system.

## 1. Introduction

Complex regional pain syndrome type I (CRPS-I) is a chronic painful condition that frequently develops after a deep tissue injury, such as a fracture or sprain, without nerve injury. CPRS-I is clinically characterized by a variety of sensory disturbances including allodynia, hyperalgesia, edema, vasomotor/sudomotor deregulation, skin and underlying tissue trophism modification. Symptoms typically begin in the distal part of the affected limb and spread to the unaffected or opposite limb [1,2,3,4,5,6,7].

The pathophysiology of this syndrome remains unclear, however inflammatory and neural mechanisms have been suggested as potential contributors. Both peripheral and central mechanisms are thought to play a prominent role; however, evidence exists indicating that oxidative stress (OS) also plays an important role [5,6,7]. Individuals with CRPS-I suffer from alterations in central and peripheral nervous system processing leading to decreased pain pressure threshold and increased temporal summation of pain [8]. These physiological changes most likely involve OS changes, which are known to be an important mechanism following tissue injury and hypoxia [9,10].

A rodent model of chronic post-ischemia pain (CPIP) was developed by Coderre et al. [1] which mimics much of the clinical symptomatology associated with CRPS-I. This model was first developed in rats [1] and later adapted for mice [11]. It produces ischemia followed by reperfusion, and its initial phase is characterized by hyperemia and edema that produces micro vascular injury, deep tissue inflammation, muscle nociceptor activation and ectopic activation of afferent sensory axons via an inflammatory cascade and endoneural ischemia [1]. Reactive oxygen species (ROS) are known to play a predominate role in the inflammatory event cascade created by prolonged hindlimb ischemia-reperfusion (IR) [1,12,13] resulting in the production of oxidants, superoxide, hydroxyl radicals and hydrogen peroxide among others. Assuming that the generation of free radicals is partially responsible for CRPS-I in CPIP, Coderre et al. [1] demonstrated that two free radical eliminators reduced signs of mechanical allodynia emphasizing the importance of oxidants in the maintenance of CRPS-I neuropathic pain symptoms. Furthermore, the presence of OS in patients with CRPS-I patients has been indirectly confirmed thereby strengthening the rationale for clinical use of antioxidants and free radical scavengers to treat and/or prevent CRPS type I [14,15].

Pain management in combination with strength and flexibility training along with manual soft tissue techniques applied to the involved extremity have been the traditional clinical treatment of CRPS [16,17,18]. In this sense, typical treatment strategies include desensitization therapy, manual therapy, progressive exercise, and patient education [16,17,18,19]. Among conservative therapies, manual therapy (i.e., joint mobilization) stands out as a possible therapeutic for the reduction of symptoms and signs of CRPS-I since it is commonly used to treat a number of painful conditions [20]. Main clinical effects of manual therapy include pain reduction, functional improvement and aspects of neurophysiological modulation [21,22]. Manual therapy is commonly used to treat a variety of musculoskeletal conditions as an adjunct treatment, but literature describing its use for managing CRPS is scarce. Clinical and preclinical studies have provided a good rationale to test the effect of manual therapy (MT) on CRPS-I and to determine the physiological contribution of oxidative stress. For example, in a clinical case series of individuals experiencing bilateral lower extremity CRPS, application of MT to the lumbar spine along with traditional conservative care resulted in meaningful clinical outcomes that were most likely associated with the MT intervention [23]. Furthermore, Kolberg et al. [24] reported that joint manipulation in humans increased catalase (CAT) activity in erythrocytes showing the antioxidative effect of manual therapy intervention.

Our research group has demonstrated that activation of inhibitory neuroreceptors such as adenosine A, opioid, cannabinoid 2 (CB_2_), peripheral/spinal and cannabinoid 1 (CB_1_) are involved in analgesic effects of MT in mice [2,25,26]. Interestingly, these endogenous systems activated by MT modulate oxidative stress. In rats, stress-activation of lipid peroxidation in plasma and liver tissue was reduced by the injection of opioid peptides while at the same time increasing catalase activity [27]. In human monocytes/macrophages, it has been shown that during inflammation the CB_1_ receptor is highly expressed and that its activation directly modulates inflammatory activity by means of production of ROS [28]. Moreover, the activation of the CB_2_ receptor may generate inhibitory signaling that directly suppresses the production of ROS stimulated by the activation of the receptor CB_1_ [29]. In addition, the adenosinergic system is known to modulate oxidative stress especially via activation of the A_1_ receptor [30].

Although the neurophysiological effects of MT has been demonstrated in other animal pain models, to date it has not been investigated in a CRPS-I model. The purpose of this study was to determine if MT can indeed reduce pain behavior and oxidative stress by means of enzymatic anti-oxidative system activation in a CRPS-I model. Thus, the results of the present study may serve as a basis for future clinical trials aiming to evaluate the effects of MT on CRPS-I or other painful conditions that have oxidative stress as the main pathophysiology. In addition, this study also shows the possibility of beneficial effects in the association of MT with anti-oxidant therapies in the treatment of chronic pain.

## 2. Materials and Methods

### 2.1. Animals

**Figure 1:**
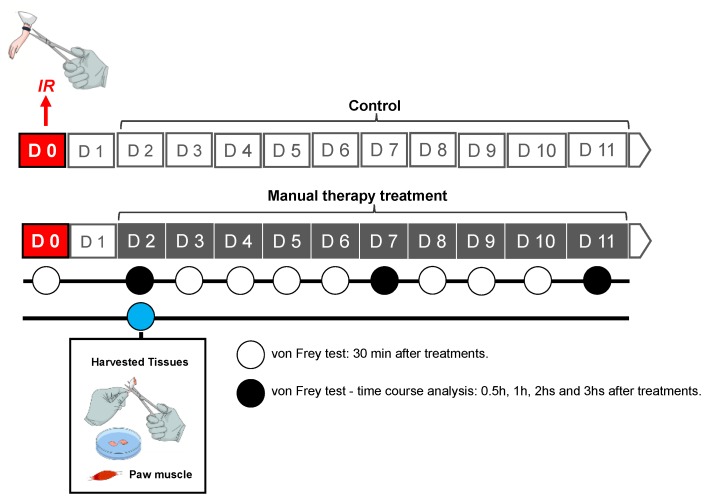
Timeline of treatment and analyses. Ischemia-reperfusion (IR): Ischemia and reperfusion; D: day; min: minutes; h: hour.

All experimental procedures were approved by the Ethics Committee of the University of Southern Santa Catarina at Palhoça, Santa Catarina, Brazil (protocol number 15.034.3.07.IV) and performed in accordance with the National Institute of Health Animal Care Guidelines (NIH publications number 80-23). Male Swiss mice (25–35 g) were obtained from the Biotério Central da Universidade Federal de Santa Catarina (UFSC, Florianópolis, Santa Catarina, Brazil) and group housed at 22 ± 2 °C under a 12 h light/12 h dark cycle (lights on at 6 a.m.) with food and water ad libitum. Mice were habituated to the testing environment for a minimum of 1 h before any experiments were conducted between 8 a.m. and noon [31]. Figure 1 shows the experimental timeline of IR injury, MT treatment and tissue harvesting.

### 2.2. Animal Model CRPS-I 

The animal model of CRPS-I was performed following experimental procedures described first for rats and later adapted for mice [11], involving exposure to prolonged hindpaw IR. This model uses an elastic O-ring (commonly used for orthodontic braces (Elástico Ligadura 000-1237, Uniden, SP, Brazil) with a 1.2-mm internal diameter placed around the right hindlimb just proximal to the ankle joint thereby producing ischemia. During this procedure, mice were anesthetized with a bolus (7%, 0.6 mL/kg, i.p.) of chloral hydrate and 20% of the initial volume at the end of the first and second hour. As previously established in rodent models, O-rings were left on the limb for 3 hours. All sham mice were subjected to the same experimental procedures except that the O-ring was slightly cut so that it only loosely surrounded the ankle so as to not occlude blood flow to the right hindpaw [32,33].

### 2.3. MT Treatment 

MT treatment was performed as previously described [25]. Mice were lightly anesthetized with 1%–2% isoflurane and the experimenter’s hand stabilized the knee joint while the ankle joint was flexed and extended to full amplitude, rhythmically with a movement frequency of approximately 40 cycles per minute. Movement frequency was performed with assistance of a metronome. Treated animals received a total of 9 minutes of MT divided in 3 series of 3 minutes each with a 30 second interval between series. Sham group animals were kept anesthetized for the same time period, with the experimenter’s hands positioned on ankle joint but no movements were performed [2,26]. Animals received daily treatments of 9-minute MT between the 2nd to 11th day after the IR procedure.

### 2.4. Mechanical Hyperalgesia

To assess mechanical hyperalgesia, mice were acclimatized to individual clear boxes (9 × 7 × 11 cm) on an elevated wire mesh platform which allowed access to the ventral hindpaw surface, as previously described [2,25]. Mechanical hyperalgesia was measured with right hindpaw stimulation in a series of 10 non-consecutive applications using calibrated 0.4 g von Frey filaments (Stoelting, Chicago, IL, USA) [26]. Results are reported as the percentage of response frequency. The time course analyses of antihyperalgesic effects caused by MT was performed at the 2nd, 7th and 11th days after the IR procedure, at 30, 60 and 90 minutes after MT treatment. In a separate set of experiments, mechanical hyperalgesia was assessed every day following MT between the 2nd to 11th day after the IR procedure.

### 2.5. Sample Collection for Biochemical Analyses 

In a separate set of experiments involving the collection of biological samples on the 2nd day after IR, all animals were euthanized 30 min after MT treatment and right hindpaw muscle tissue samples were surgically harvested. The tissues were weighed and homogenized in 10 volumes (1:10, *w*/*v*) of ice-cold 0.1 M phosphate buffer (pH 7.4). To discard cell debris and nuclei, homogenates were centrifuged at 750× *g* for 10 min at 4 °C. After discarding the pellet, aliquots of supernatants were separated and used for determination of oxidative stress parameters.

### 2.6. Determination of Oxidative Stress and Antioxidant Enzymes Levels

Thiobarbituric acid reactive species (TBARS) formation was measured during an acid-heating reaction [34]. Samples were heated for 15min in a boiling water bath, mixed with 1ml of trichloroacetic acid (TCA) 10% and 1ml of thiobarbituric acid 0.67%. TBARS was determined by the absorbance at 535nm. Results are expressed as malondialdehyde (MDA) equivalents (nmol/mg protein).

Oxidative damage to proteins was measured by determining the carbonyl groups based on the reaction with dinitrophenylhydrazine (DNPH) [35]. Precipitation of proteins were conducted by the addition of 20% trichloroacetic acid and redissolved in DNPH with the absorbance read at 370 nm. Results were reported as nmol of carbonyl content per mg of protein (nmol/mg protein).

Catalase (CAT) activity was measured by the rate of decrease in hydrogen peroxide absorbance at 240 nm [36]. Briefly, hindpaw tissue samples were sonicated in 50nmol/l phosphate buffer (pH 7.0), and the resulting suspension was centrifuged at 3000 g for 10 min. The supernatant was used for the enzyme assay. Results were reported as units per milligram of protein (U/mg protein).

The activity of superoxide dismutase (SOD) was determined by measuring the inhibition of adrenaline auto-oxidation spectrophotometrically at 480 nm [37] and was represented as units per milligram of protein (U/mg protein).

Bovine albumin was used as a standard to normalize all biochemical measurements [38].

### 2.7. Mitochondrial Function Analyses

Nicotinamide adenine dinucleotide (NADH)-dependent ferricyanide reduction was used to measure Complex I activity [39]. Samples were coupled with reagents 100mM potassium phosphate buffer, 10 mM ferricyanide, 14 mM NADH and 2 mM rotenone, and analyzed at 420 nm by a spectrophotometer with readings taken minute by minute for a total of 3 minutes.

The 2,6-diclorophenolindophenol (DCPIP) reduction was used to measure Complex II activity as described by Fisher et al. [40]. The tissue sample was incubated for 20 minutes at 30 °C water bath with 62.5 mM potassium phosphate buffer, 250 mM sodium succinate and 0.5 mM DCPIP. After incubation, 100 mM sodium azide, 2 mM rotenone and 0.5 mM DCPIP were added and then minute by minute spectrophotometric readings taken for a total of 5 minutes at 600 nm.

Complex IV activity was determined by calculating the absorbance reduction caused by reduced cytochrome c oxidation as described by Rustin et al. [41]. In the incubation environment, 62.5 mM potassium phosphate buffer, 125 mM lauryl maltoside was added and sample diluted with SETH buffer (Sucrose, EDTA, Tris and Heparine) and 1% cytochrome c. Analyses were performed at 550 nm by a spectrophotometer with readings taken minute by minute for 10 min. The results of mitochondrial function were expressed in nmol/min by mg of protein.

### 2.8. Statistical Analysis

The Shapiro–Wilk test was used to evaluate the normality assumption of all behavioral and biochemical data. All variables in the present study showed a normal distribution. Differences among experimental groups were determined by one or two-way ANOVA followed by Student–Newman–Keuls Multiple Comparison or Bonferroni post hoc test, respectively, as appropriate. A value of *p* < 0.05 was considered to be statistically significant. All data are presented as mean (standard deviation, SD).

## 3. Results

**Figure 2:**
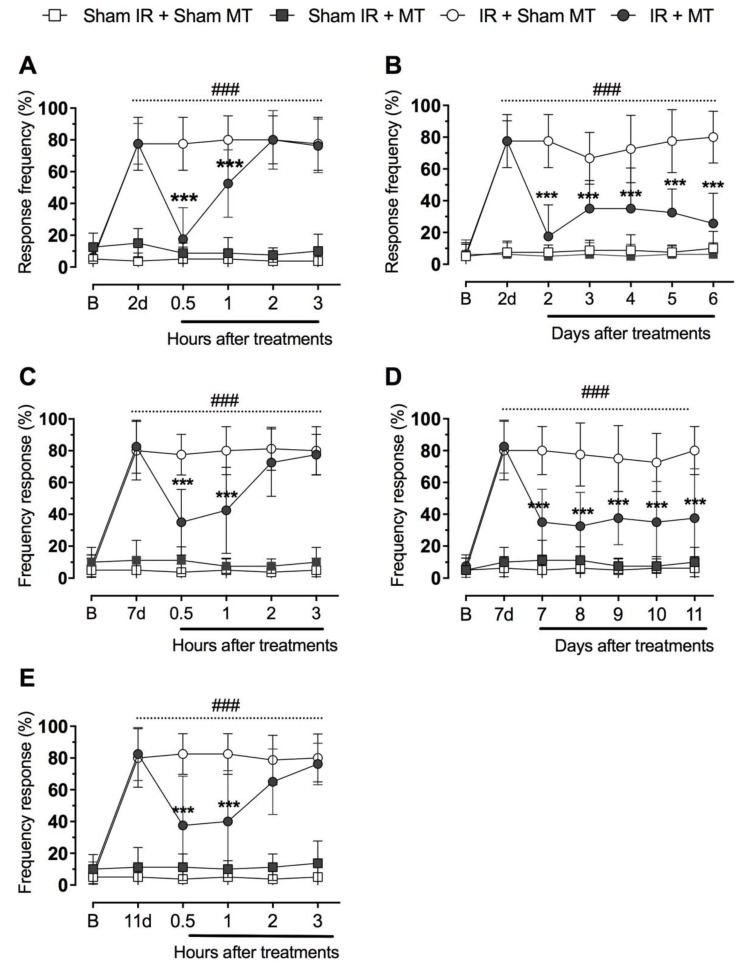
Effect of manual therapy (MT) on mechanical hyperalgesia. Time course analysis at the 2nd day (panel **A**). Daily treatment with 9-minute ankle joint mobilization between the 2nd to 6th day after IR procedure (panel **B**). Time course analysis at 7th day (panel **C**). Daily treatment with 9-minutes of MT between the 7th to 11th day after IR procedure (panel **D**). Time course analysis at 11th day (panel **E**). Each point represents the mean of 8 animals and vertical lines show the SD. Statistical analyses were performed by two-way ANOVA followed by Bonferroni test. The symbols denote a significant difference of *** *p* < 0.001 when compared to IR + Sham MT group or ^###^ *p* < 0.001 when compared to Sham + Sham MT group. MT: Manual therapy, IR: Schemia and reperfusion.

Figure 2 shows that mice hindpaw IR induced marked and long-lasting mechanical hyperalgesia, as observed by the enhancement of the response frequency to the von Frey filament application in comparison to sham mice (*p* = 0.001) (Figure 2A–E). We observed that the acute MT treatment (IR + MT group) on the 2nd, 7th and 11th days after IR reduced mechanical hypersensitivity induced by IR. Significant differences between groups (IR + Control vs IR + MT) were observed at 0.5 h (*p* = 0.001) and 1 h (*p* = 0.001) after MT (Figure 2A,C,E). Furthermore, the repeated daily MT treatments (2–7 or 7–11 days) decreased (*p* = 0.001) the mechanical hypersensitivity induced by IR when assessed 30 minutes after MT (Figure 2B,D).

**Figure 3:**
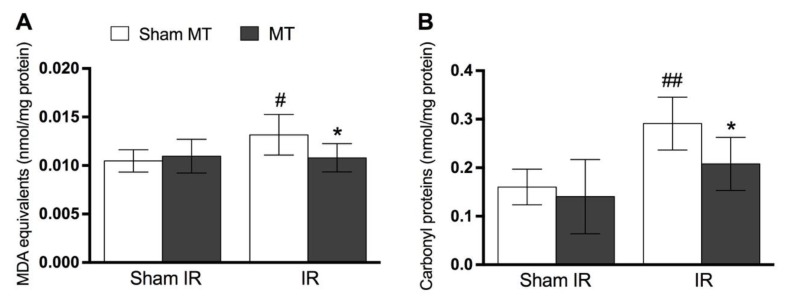
Evidence of the effects of MT on oxidative stress markers at the 2nd day after the IR procedure. Panels show the preventive effect of MT on the increase of MDA (panel **A**) and carbonyl proteins (panel **B**). Each point represents the mean of 8 animals and vertical lines show the SD. Statistical analyses were performed by one-way ANOVA followed by Newman–Keuls Multiple Comparison Test. The symbols denote a significant difference of * *p* < 0.05 when compared to IR + Sham MT group, # *p* < 0.05 or ^##^ *p* < 0.001 when compared to Sham + Sham MT group. MT: Manual therapy, IR: Ischemia and reperfusion, MDA: Malondialdehyde.

Figure 3 shows that at the 2nd day after IR injury, the concentrations of MDA (Figure 3A, *p* = 0.02) and protein carbonyls (Figure 3B, *p* = 0.01) in muscle paw tissue were increased compared to Sham and Sham + MT groups. MT significantly prevented MDA (Figure 3A, *p* = 0.03) and carbonyls protein increase (Figure 3B, *p* = 0.03). 

**Figure 4:**
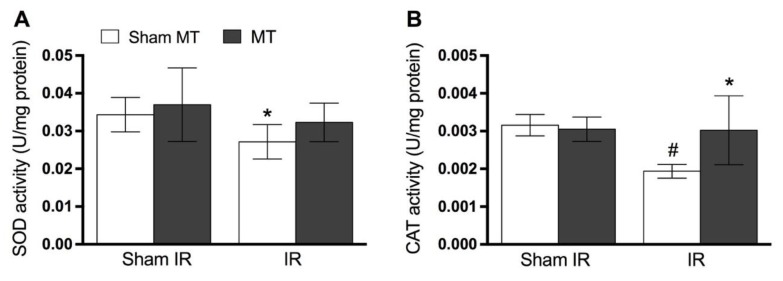
Evidence of MT effects on anti-oxidant enzymes levels at the 2nd day after IR procedure. Panel **A** shows that there was no significant difference on superoxide dismutase (SOD) activity, while panel **B** shows a significant difference on CAT activity. Each point represents the mean of 8 animals and vertical lines show the SD. Statistical analyses were performed by one-way ANOVA followed by Newman–Keuls Multiple Comparison Test. The symbols denote a significant difference of * *p* < 0.05 when compared to IR + Sham MT group, # *p* < 0.05 when compared to Sham + Sham MT group. MT: Manual therapy, IR: Ischemia and reperfusion, SOD: Superoxide dismutase, CAT: Catalase.

Figure 4 shows that IR injury diminishes the levels of SOD (Figure 4A, *p* = 0.03) and CAT (Figure 4B, *p* = 0.02) activity in the animals’ paw tissue on day 2 following IR. MT treatment effectively prevented the decrease in the activity of CAT (Figure 4B, *p* = 0.02), but not SOD (Figure 4A, *p* = 0.31) induced by IR. 

**Figure 5:**
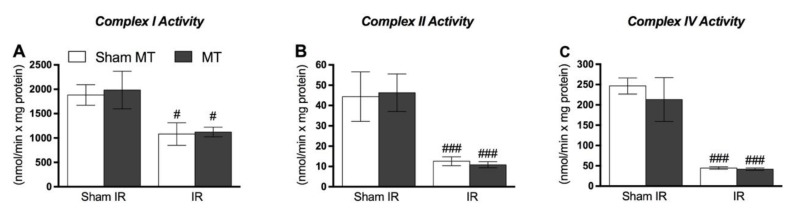
Effects of MT on mitochondrial function at the 2nd day after IR procedure. Complex I activity (**A**), Complex II activity (**B**) and complex IV activity (**C**) all panels show that IR reduces mitochondrial function but ankle joint mobilization could not prevent the mitochondrial function reduction. Each point represents the mean of 8 animals and vertical lines show the SD. Statistical analyses were performed by One-way ANOVA followed by Newman–Keuls multiple comparison test. The symbols denote a significant difference of # *p* < 0.05 or ### *p* < 0.001 when compared to Sham + Sham MT group. MT: Manual therapy, IR: Ischemia and reperfusion.

Figure 5 shows that IR injury diminishes Complex I (*p* = 0.001), II (*p* = 0.001) and IV activity (*p* = 0.001) in the animal hindpaw tissue on day 2 following IR. However, MT treatment was ineffective in preventing decreases in mitochondrial complex activity. 

## 4. Discussion

The results of this study show that MT produces analgesic and anti-oxidative effects in a murine model of CRPS-I. Our findings further show that the MT reduced mechanical hyperalgesia in all days evaluated, prevented the increase of TBARS and protein carbonyls concentrations, and prevented the reduction of CAT activity, while not influencing SOD activity. No effects from MT were observed in mitochondrial complex activity. The effect of MT has been demonstrated in multiple nerve injury models, such as the sciatic nerve crush injury model [42], postoperative pain model, and plantar incision surgery model [2], in which ankle joint mobilization produced an analgesic effect. The present set of experiments demonstrated for the first time an analgesic effect of MT in a murine CRPS-I model. 

While CRPS pathophysiology is not fully understood, oxidative events are thought to give rise to primary afferent nociceptor sensitization which contributes to central sensitization. It has been well documented that prolonged hindlimb IR produces a subsequent cascade of inflammatory events, with pivotal roles being played by reactive oxygen species [12,13]. Ischemia-reperfusion results in production of oxidants, superoxide, hydroxyl radicals hydrogen peroxide, among other ROS initiated by the enzymes NADPH oxidase [43,44] or xanthine oxidase [45,46]. Coderre et al. [1] demonstrated that free radical scavengers reduced CPIP symptoms thereby emphasizing the important role that oxidants play in the maintenance of neuropathic pain-like symptoms in CRPS-I models [47]. Thus, the observed anti-oxidative effects of MT may be associated with the analgesia induced by MT in this current neuropathic pain model. 

We observed that the IR procedure that induced mechanical hyperalgesia was maintained up to the 11th day of evaluation and that acute MT treatment was able to reduce mechanical hyperalgesia for 1h after treatment. Repeated treatments did not show a cumulative effect, since after MT treatments an increase in duration of analgesia was not observed. The specific analgesic mechanisms underlying peripheral joint mobilization remain unclear, but activation of inhibitory neuroreceptors such as opioid, cannabinoid 1(CB_1_) and 2 (CB_2_) receptors are all thought to play a role [2,25,26]. 

Possible explanations for the MT-related findings in the current study are the effects of the neuroreceptors (opioid, cannabinoid and adenosine receptors) activated by the oxidative system. In this context, it has been shown that the injection of opioid peptides in rats decrease the stress-induced activation of lipid peroxidation in plasma and liver tissue as well as increase catalase activity [27]. Interestingly, it has been shown in human monocytes/macrophages that during inflammation the CB_1_ receptor is highly expressed and that its activation directly modulates inflammatory activity by means of production of ROS [28]. Conversely, CB_2_ receptor activation exerts an anti-inflammatory response, such as inhibition of chemotactic movement in response to monocyte chemoattractant protein-1 (MCP-1) [48]. Moreover, the activation of CB_2_ receptor may generate inhibitory signaling and directly suppress the production of ROS stimulated by the activation of the receptor CB_1_ [29].

Recently, our research group has shown that MT reduces post-operative pain in mice by activation of the CB_2_, but not the CB_1_ receptor. That led us to believe that in the current study, the observed oxidative stress reduction (MDA and protein carbonyls) is due to the inhibitory effect on the activation of the CB_2_ receptor mediated by MT on the ROS production stimulated by the activation of the CB_1_ receptor.

The adenosinergic system also has been shown to modulate oxidative stress especially on activation of the A_1_ receptor. The antioxidant effect of an adenosine A_1_ receptor agonist cyclopentyladenosine (CPA) was recently studied in a focal cerebral ischemia model. Changes in lipid peroxidation (LPO) processes in the brain and blood tissue were demonstrated following ischemic brain injury. Changes in the ratio between LPO and antioxidant protection were less pronounced after cyclopentyladenosine treatment [30]. Current thought is that signaling activated by adenosine and/or other receptors (such as opioid or bradykinin) converge on key targets like mitochondrial K_ATP_ channels or the mitochondrial permeability transition pore (MPTP) [49,50,51,52]. The MPTP may be inhibited through control of protein phosphorylation (together with effects of K_ATP_ opening), or by inhibition of cellular oxidative stress and subsequent MPTP thiol modification [49,52]. Moreover, it has been shown that oxidative stress is selectively modulated endogenously by the A_1_ receptor in ischemic hearts [52].

In parallel of this literature, Martins et al. [2] verified that MT reduces mechanical hyperalgesia induced by plantar incision surgery and these effects were mediated by the activation of A_1_ receptors activation. Therefore, we may consider the hypothesis that in the present study, endogenous adenosine might have been secreted during MT which mediated analgesia and oxidative stress reduction. We found that ankle joint mobilization significantly reduced oxidative damage in the hindpaw, potentially suggesting a novel analgesic mechanism of MT by increased CAT activity in a CRPS-I model. These findings corroborate the study of Kolberg et al. [24] in humans, where they also observed that joint manipulation increased CAT activity in erythrocytes showing an anti-oxidative effect of manual therapy. In contrast to our findings, they did not find changes in lipidic peroxidation concentrations. These discrepancies may be explained by differences in the analyzed tissues/cells and/or the particular models used. The results of the present study are important in the clinical setting, since MT (joint mobilization) is widely used in the rehabilitation protocols of patients with chronic pain. In this sense, our findings suggest that MT may be used to treat CRPS-1 in humans, since it has an anti-oxidant effect. In addition, these results support the need for future clinical trials that associate MT with anti-oxidant therapies for the effective treatment of CRPS-I.

### Limitations

This study did not evaluate the effect of MT on oxidative stress at other (non-peripheral) pain modulation sites such as the spinal cord, brainstem or sensory cortex which would allow a broader understanding of the effects of MT on CRPS-I. This study also did not analyze the oxidative stress parameters in the blood of mice, which would be interesting to investigate in a clinical setting. Future studies are needed to improve our understanding regarding the association between oxidative stress and the antihyperalgesic effects caused by MT and to establish the precise neurobiological systems underlying this effect of MT on oxidative stress parameters.

## 5. Conclusions

In summary, current results extended previous findings and demonstrated that daily sessions of MT presented antihyperalgesic effects mediated, at least in part, through (1) prevention of TBARS and protein carbonyls increase in peripheral (hindpaw) tissue and, (2) improvement of the antioxidant defense system (increase of CAT, but not SOD activity). MT did not change the analyzed mitochondrial complex activity. Together, these new findings contribute to a better understanding of the neurobiological mechanisms responsible for the therapeutic effect of MT, as well as provide additional support for its use as adjunctive treatment of CRPS-I.
